# Factors Determining Kinesin Motors in a Predominant One-Head-Bound or Two-Heads-Bound State During Its Stepping Cycle

**DOI:** 10.3390/biom15050717

**Published:** 2025-05-13

**Authors:** Xiao-Xuan Shi, Yu-Ru Liu, Ping Xie

**Affiliations:** 1 School of Pharmaceutical Engineering, Chongqing Chemical Industry Vocational College, Chongqing 401220, China; xxshi2025@gmail.com; 2 Laboratory of Soft Matter Physics, Institute of Physics, Chinese Academy of Sciences, Beijing 100190, China; yrliu@aphy.iphy.ac.cn

**Keywords:** kinesin, stepping manner, one-head-bound state, two-heads-bound state

## Abstract

At physiological or saturating ATP concentrations, some families of kinesin motors, such as kinesin-1 and kinesin-2, exhibit a predominant two-heads-bound (2HB) state during their stepping cycle on microtubules, while others, such as kinesin-3, exhibit a predominant one-head-bound (1HB) state. An interesting but unclear issue is what factors determine a kinesin motor in the predominant 1HB and 2HB states. Here, on the basis of the general chemomechanical pathway of the kinesin motors, a theory is given on fractions of 1HB and 2HB states. With the theory, the factors affecting a kinesin motor in the predominant 1HB and 2HB states are determined. The results about the effects of ATP concentration, ADP concentration and external load on the fractions of 1HB and 2HB states are presented. Furthermore, the theory is applied to kinesin-1, kinesin-2, kinesin-3, kinesin-5 and kinesin-13 motors, with the theoretical results agreeing well with published experimental data.

## 1. Introduction

The kinesin superfamily can be categorized into 14 subfamilies according to their structural similarity and function [1,2,3,4]. Most subfamilies of the kinesin motors are in a dimeric form, containing the two motor domains (also called heads) that are connected together by a common and long stalk via their flexible neck linkers (NLs) [5,6]. By making use of the chemical energy derived from the hydrolysis of ATP molecules, the dimeric motors can move processively on microtubules (MTs) toward the plus end, carrying out multiple cellular functions that include cargo transport [1,2,3,4]. It was well determined that the dimer can move along an MT filament in a hand-over-hand manner and alternate between a one-head-bound (1HB) state and a two-head-bound (2HB) state [7,8,9,10,11,12,13,14,15,16,17]. Interestingly, it was found that under saturating or physiological ATP concentrations, some families of kinesin motors, such as kinesin-1, kinesin-2 and kinesin-5, exhibit the predominant 2HB state [13,14,15,16], while others, such as kinesin-3, exhibit the predominant 1HB state [17]. It was generally believed that adopting the predominant 2HB state would ensure the kinesin-1 and kinesin-2 dimers have high processivity [14] and make the kinesin-5 motors able to generate large forces as teams in anaphase spindle elongation [18,19]. By contrast, adopting the predominant 1HB state could make the stepping dimer have a small effect on the removal of tubulin heterodimer from the MT lattice because in the 1HB state no internal force is present between the two heads, which arises from the NL stretching [20,21,22,23,24,25]. Additionally, studies indicated that on the MT lattice, instead of directional movement kinesin-13 MCAK dimer makes an unbiased one-dimensional diffusion in the predominant 1HB state [26,27]. For kinesin-1, the effect of ATP concentration on the duration of the 2HB state and that of the 1HB state in a stepping cycle were also studied both experimentally and theoretically [11,13,14,28,29,30], and, in particular, the controversial experimental data [11,13,28] were explained consistently [30].

What are the factors that determine the motor in the predominant 1HB or 2HB state? How does ATP concentration affect the kinesin-3 motor in the predominant 1HB or 2HB state? How does the external load affect a kinesin motor in the predominant 1HB or 2HB state? If free ADP molecules are present in the solution, how does ADP concentration affect a kinesin motor in the predominant 1HB or 2HB state? To address the unclear issues, on the basis of the general chemomechanical pathway of the kinesin motor, a theory is given on the fraction of the 1HB or 2HB state. With the theory, the factors that affect a kinesin dimer in the predominant 1HB or 2HB state are determined, and the effects of ATP concentration, external load and ADP concentration on the 1HB and 2HB states are studied. Furthermore, the theory is applied for kinesin-1, kinesin-2, kinesin-3, kinesin-5 and kinesin-13 motors, explaining well the available experimental data.

## 2. General Chemomechanical Pathway of Kinesin Dimer

**Figure 1:**
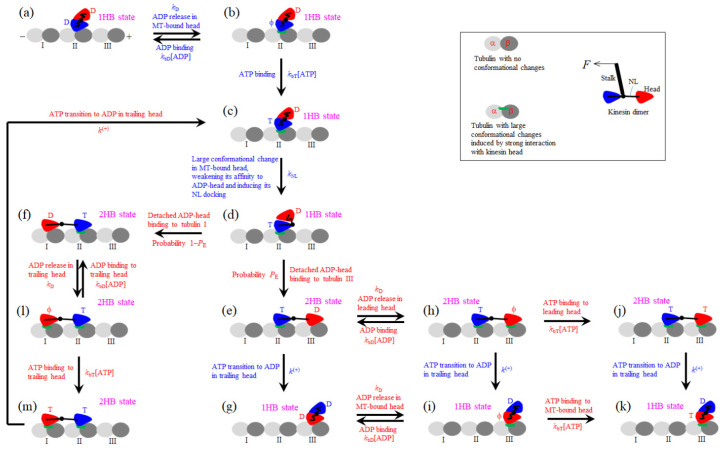
General chemomechanical pathway of the kinesin motor. T represents ATP and ADP. Pi, D represents ADP, and ϕ represents nucleotide-free. The inset shows schematically the normal tubulin with no large conformational changes, the tubulin with the large conformational changes induced by the strong binding of the kinesin head in the ϕ or T state to the tubulin, and the structural components of the kinesin dimer. For clarity, only in the inset is the motor’s stalk shown. Stages (**a**–**m**) state transitions (see Section 2 for detailed descriptions). After the dimer in the ADP state binds to MT (**a**), ADP is released from the MT-bound head (**b**), followed by ATP binding (**c**). Then, the transitions from stage (**c**) through stage (**k**), which include the futile coupling transitions from stage (**d**) to (**m**) and returning to stage (**c**), correspond to a stepping cycle of the motor. The mechanical steps include the diffusion of the ADP-head from the local tubulin to the intermediate (INT) position, i.e., the transition from stage (**e**) to (**g**), from stage (**h**) to (**i**), from stage (**j**) to (**k**), or from stage (**m**) to (**c**), which occurs after ATP transition to ADP in the TH, and the diffusion of the detached ADP-head from the INT position to the nearest tubulin, i.e., the transition from stage (**d**) to either stage (**e**) or (**f**), which occurs after the decrease in the binding energy of the MT-bound ATP-head to the detached ADP-head. The other transitions correspond to the ATPase activity and the large conformational change associated with the NL docking of the head. Since the rates of the mechanical steps (on the order of 1 μs^−1^) are much larger than the rate constants of the ATPase activity and the large conformational change in the ATP head (on the order of 1~10 ms^−1^), the rates of the mechanical steps are not indicated.

The pathway for the chemomechanical coupling of the kinesin dimer is shown schematically in Figure 1, which is modified from that presented before [30]. Here, besides free ATP molecules, we also consider that free ADP molecules are present in solution. The main elements on which the pathway is constructed are presented in Section S1 (see Supplementary Information, SI). Since for most families of kinesin motors the rate of ATP transition to ADP in the leading head (LH) is much smaller than that in the trailing head (TH), for simplicity, we do not consider ATP transition to ADP in the LH. Since this work focuses on the mean durations and fractions of the 1HB and 2HB states in a mechanical step, the dissociation of the motor from MT is not considered.

Initially, one head of the dimer in the ADP state binds with weak affinity *E*_w2_ (defined in SI) to tubulin II on an MT filament (Figure 1a), where the two heads have a high binding energy to each other, preventing the detached head from diffusing far away from the MT-bound head and from binding to the nearest unoccupied tubulin I or III. After ADP release from the MT-bound head (Figure 1b), either ATP or ADP can bind. After ATP binds (Figure 1c), a large conformational change in the ATP-head, together with the docking of its NL and the great decrease in its binding energy to the detached ADP-head, occurs (Figure 1d). Then, the detached ADP-head can, with a probability *P*_E_, diffuse rapidly forward and bind to tubulin III with affinity *E*_w2_ (Figure 1e) and, with probability 1–*P*_E_, diffuse backward and bind to tubulin I (Figure 1f) (noting that the latter requires overcoming the energy barrier arising from the NL docking and the large conformational change in the ATP-head).

In Figure 1e, before ADP release from the LH, ATP transition to ADP in the TH can occur. Due to the large force on the TH, which arises from the NL stretching, and the weak affinity of *E*_w1_ (with *E*_w1_ << *E*_w2_, see SI) of the TH to the local tubulin II, the TH detaches and diffuses to the intermediate (INT) position, where the detached ADP-head has the high binding energy to the MT-bound head (Figure 1g). In Figure 1e, before ATP transitions to ADP in the TH, ADP release from the LH can occur (Figure 1h). In Figure 1g, ADP release from the MT-bound head occurs (Figure 1i). In Figure 1h, before either ATP or ADP binds to the LH, ATP transition to ADP in the TH can occur. The TH detaches and diffuses to the INT position, where the two heads have the high binding energy to each other (Figure 1i). In Figure 1h, before ATP transitions to ADP in the TH, either ATP or ADP can bind to the LH (Figure 1j or Figure 1e). In Figure 1j, after ATP transitions to ADP in the TH, the TH detaches and diffuses to the INT position, where the two heads have the high binding energy to each other (Figure 1k). In Figure 1i, either ATP or ADP can bind to the nucleotide-free MT-bound head (Figure 1k or Figure 1g). The state in Figure 1k is the same as that in Figure 1c, except that in Figure 1k the motor takes a forward step.

In Figure 1f, after ADP release from the TH (Figure 1l), either ATP or ADP can bind to the TH (Figure 1m or Figure 1f). In Figure 1m, after ATP transitions to ADP in the TH, the TH detaches and diffuses to the INT position, with the system returning to Figure 1c.

As it is seen, a stepping cycle corresponds to the transitions from Figure 1c through Figure 1k (including the futile coupling transitions from Figure 1d to Figure 1m and returning to Figure 1c). The mechanical steps include the diffusion of the ADP-head from the local tubulin to the INT position (i.e., the transition from Figure 1e to Figure 1g, from Figure 1h to Figure 1i, from Figure 1j to Figure 1k, or from Figure 1m to Figure 1c), which occurs after ATP transition to ADP in the TH, and the diffusion of the detached ADP-head from the INT position to the nearest tubulin (i.e., the transition from Figure 1d to either Figure 1e or Figure 1f), which occurs after the decrease in the binding energy of the MT-bound ATP head to the detached ADP-head. The other transitions correspond to the ATPase activity and the large conformational change in the head. As shown before [31], in the optical trapping assay with the movable trap, the load on the stalk of the motor has little effect on the detached head diffusing from the rear position to the INT position and has a large effect on the detached head diffusing from the INT position to the front/rear tubulin.

## 3. Results and Discussion

### 3.1. General Theory on Fractions of 1HB and 2HB States

In this section, we provide the general theory on fractions of 1HB and 2HB states of the kinesin dimer on the basis of the pathway shown in Figure 1, where, besides free ATP molecules, we also consider the presence of free ADP molecules in solution. We also consider a backward load, *F*, on the stalk of the motor, as in the optical trapping experiments using the movable trap.

The probability *P*_E_, as defined in Figure 1, has the following form [31]:

(1) $$P_{E}=\frac{\exp\left(\frac{E_{0}-F\delta }{k_{B}T}\right)}{\exp\left(\frac{E_{0}-F\delta }{k_{B}T}\right)+1},$$

where *k*_B_ is the Boltzmann constant, *T* the absolute temperature, *E*_0_ the energy change in the large conformational change in the head and NL docking induced by ATP binding, and δ the load-sensitivity distance for the movement of the detached ADP-head from the INT position to the nearest unoccupied tubulin.

On the basis of Figure 1, in a stepping cycle the factions of the motor in the 1HB and 2HB states, which are denoted by *F*_1HB_ and *F*_2HB_, respectively, can be derived as follows (see Section S2 in Supplementary Materials):

(2) $$F_{1\mathrm{HB}}=\frac{A_{1}+A_{3}+A_{5}}{1+A_{1}+A_{2}+A_{3}+A_{4}+A_{5}+A_{6}+A_{7}+A_{8}},$$

(3) $$F_{2\mathrm{HB}}=\frac{1+A_{2}+A_{4}+A_{6}+A_{7}+A_{8}}{1+A_{1}+A_{2}+A_{3}+A_{4}+A_{5}+A_{6}+A_{7}+A_{8}},$$

(4) $$\frac{F_{2\mathrm{HB}}}{F_{1\mathrm{HB}}}=\frac{1+A_{2}+A_{4}+A_{6}+A_{7}+A_{8}}{A_{1}+A_{3}+A_{5}},$$

(5) $$A_{1}=\frac{\alpha \left(\gamma_{T}[\mathrm{ATP}]+\gamma_{D}[\mathrm{ADP}]+\alpha \right)+\gamma_{T}[\mathrm{ATP}]+\alpha }{P_{E}\beta \left(\gamma_{T}[\mathrm{ATP}]+\gamma_{D}[\mathrm{ADP}]+\alpha \right)},$$

(6) $$A_{2}=\frac{\left(1-P_{E}\right)}{P_{E}}\frac{\left(\gamma_{T}[\mathrm{ATP}]+\gamma_{D}[\mathrm{ADP}]\right)\left(\alpha +1\right)}{\gamma_{T}[\mathrm{ATP}]}-\frac{\left(1-P_{E}\right)}{P_{E}}\frac{\gamma_{D}[\mathrm{ADP}]\left(\gamma_{T}[\mathrm{ATP}]+\gamma_{D}[\mathrm{ADP}]\right)}{\gamma_{T}[\mathrm{ATP}]\left(\gamma_{T}[\mathrm{ATP}]+\gamma_{D}[\mathrm{ADP}]+\alpha \right)},$$

(7) $$A_{3}=\alpha +\frac{\alpha \gamma_{D}[\mathrm{ADP}]\left(\gamma_{T}[\mathrm{ATP}]+\gamma_{D}[\mathrm{ADP}]+\alpha +1\right)}{\gamma_{T}[\mathrm{ATP}]\left(\gamma_{T}[\mathrm{ATP}]+\gamma_{D}[\mathrm{ADP}]+\alpha \right)},$$

(8) $$A_{4}=\frac{1}{\left(\gamma_{T}[\mathrm{ATP}]+\gamma_{D}[\mathrm{ADP}]+\alpha \right)},$$

(9) $$A_{5}=\frac{\alpha \left(\gamma_{T}[\mathrm{ATP}]+\gamma_{D}[\mathrm{ADP}]+\alpha +1\right)}{\gamma_{T}[\mathrm{ATP}]\left(\gamma_{T}[\mathrm{ATP}]+\gamma_{D}[\mathrm{ADP}]+\alpha \right)},$$

(10) $$A_{6}=\frac{\gamma_{T}[\mathrm{ATP}]}{\alpha \left(\gamma_{T}[\mathrm{ATP}]+\gamma_{D}[\mathrm{ADP}]+\alpha \right)},$$

(11) $$A_{7}=\frac{\left(1-P_{E}\right)}{P_{E}}\frac{\left(\alpha +1\right)}{\gamma_{T}[\mathrm{ATP}]}-\frac{\left(1-P_{E}\right)}{P_{E}}\frac{\gamma_{D}[\mathrm{ADP}]}{\gamma_{T}[\mathrm{ATP}]\left(\gamma_{T}[\mathrm{ATP}]+\gamma_{D}[\mathrm{ADP}]+\alpha \right)},$$

(12) $$A_{8}=\frac{\left(1-P_{E}\right)}{P_{E}}\frac{\left(\alpha +1\right)}{\alpha }-\frac{\left(1-P_{E}\right)}{P_{E}}\frac{\gamma_{D}[\mathrm{ADP}]}{\alpha \left(\gamma_{T}[\mathrm{ATP}]+\gamma_{D}[\mathrm{ADP}]+\alpha \right)},$$

where $\alpha =k^{\left(+\right)}/k_{D}$ and $\beta =k_{\mathrm{NL}}/k_{D}$ are dimensionless constants, $\gamma_{T}=k_{\mathrm{bT}}/k_{D}$ and $\gamma_{D}=k_{\mathrm{bD}}/k_{D}$ are constants in units of μM^−1^, *k*^(+)^ is the rate of ATP transition to ADP in the head with the forward NL orientation, *k*_D_ is the rate of ADP release from the MT-bound ADP-head, *k*_NL_ is the rate of the large conformational change in the ATP-head in the 1HB state together with its NL docking and reduction in the binding energy between the two heads, $k_{\mathrm{bT}}$ is the second-order ATP-binding rate to the nucleotide-free head, [ATP] is the ATP concentration, $k_{\mathrm{bD}}$ is the second-order ADP-binding rate to the nucleotide-free head, and [ADP] is the ADP concentration. Rates *k*^(+)^, *k*_D_, $k_{\mathrm{bT}}$ and $k_{\mathrm{bD}}$ are independent of the force on the NL, which is consistent with the available experimental data [32,33,34,35] (see Section S1). As noted before [31], under the backward load, rate *k*_NL_ is also independent of the backward load. Thus, α, β, $\gamma_{T}$ and $\gamma_{D}$ are independent of the backward load. For clarity, the meanings of α, β, $\gamma_{T}$ and $\gamma_{D}$ are explained in Table 1.

On the basis of Figure 1, the duration of a stepping cycle (i.e., the transitions from Figure 1c through Figure 1k, including the futile coupling transitions from Figure 1d to Figure 1m and returning to Figure 1c) can be derived as follows (see Section S3 in SI):

(13) $$\tau =\frac{1+A_{1}+A_{2}+A_{3}+A_{4}+A_{5}+A_{6}+A_{7}+A_{8}}{\gamma_{T}[\mathrm{ATP}]A_{5}+\alpha A_{6}}\frac{1}{k_{D}}.$$

### 3.2. Fractions of 1HB and 2HB States Under Saturating ATP Concentrations, No Free ADP and No Load

In this section, we consider saturating ATP concentrations, with $1/\left(\gamma_{T}\left[\mathrm{ATP}\right]\right)$ approaching 0, no free ADP, with [ADP] = 0, and no load on the motor, with *F* = 0. As shown before for kinesin-1, kinesin-2 and kinesin-3 dimers [31,36], *P*_E_ approaches one when *F* = 0. Thus, we take *P*_E_ = 1 in this section. With $1/\left(\gamma_{T}\left[\mathrm{ATP}\right]\right)$ = 0, [ADP] = 0 and *P*_E_ = 1, from Equations (3)–(12) we have the following:

(14) $$\frac{F_{2\mathrm{HB}}}{F_{1\mathrm{HB}}}=\frac{\beta \left(1+\alpha \right)}{\alpha \left(1+\alpha +\alpha \beta \right)}.$$

**Figure 2:**
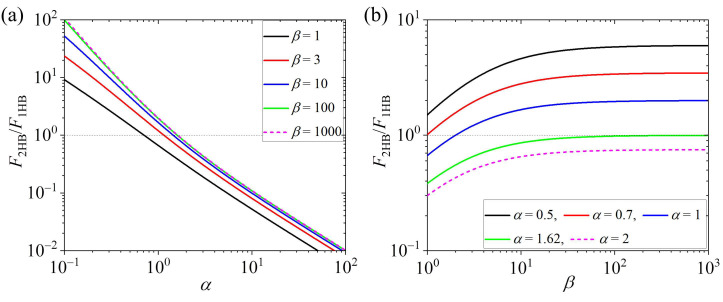
Ratio of the fraction of 2HB state to that of 1HB state, $F_{2\mathrm{HB}}/F_{1\mathrm{HB}}$, under saturating ATP concentrations, no load and no free ADP. Under this condition, $F_{2\mathrm{HB}}/F_{1\mathrm{HB}}$ is determined mainly by $\alpha =k^{\left(+\right)}/k_{D}$ and mildly by $\beta =k_{\mathrm{NL}}/k_{D}$. Thin dotted lines correspond to $F_{2\mathrm{HB}}/F_{1\mathrm{HB}}$ = 1, at which the 1HB and 2HB states have the same fraction. When $F_{2\mathrm{HB}}/F_{1\mathrm{HB}}$ > 1, the motor is in the predominant 2HB state, and when $F_{2\mathrm{HB}}/F_{1\mathrm{HB}}$ < 1, the motor is in the predominant 1HB state. (**a**) $F_{2\mathrm{HB}}/F_{1\mathrm{HB}}$ vs. α for various values of β. (**b**) $F_{2\mathrm{HB}}/F_{1\mathrm{HB}}$ vs. β for various values of α.

From Equation (14), it is noted that the motor in the predominant 2HB or 1HB state is determined by only two parameters. One is $\alpha =k^{\left(+\right)}/k_{D}$, the ratio of the rate of ATP transition to ADP in the TH to the rate of ADP release from the MT-bound head. The other one is $\beta =k_{\mathrm{NL}}/k_{D}$, the ratio of the rate of NL docking of the MT-bound ATP-head in the 1HB state to the rate of ADP release from the MT-bound head. Considering that the NL docking occurs fast [37], it is expected that *k*_NL_ is greater than or equal to *k*_D_. Thus, in this work we take $\beta =k_{\mathrm{NL}}/k_{D}$≥ 1. Using Equation (14), the calculated results of $F_{2\mathrm{HB}}/F_{1\mathrm{HB}}$ vs. α for various values of β are shown in Figure 2a, and those of $F_{2\mathrm{HB}}/F_{1\mathrm{HB}}$ vs. β for various values of α are shown in Figure 2b.

From Figure 2a, it is seen that $F_{2\mathrm{HB}}/F_{1\mathrm{HB}}$ changes sensitively with α. At large, β > 100, $F_{2\mathrm{HB}}/F_{1\mathrm{HB}}$ is independent of β and is determined solely by α (with $F_{2\mathrm{HB}}/F_{1\mathrm{HB}}=\left(\alpha +1\right)/\alpha^{2}$ at β→∞). At β > 100, if α < 1.62, the motor is in the predominant 2HB state, with $F_{2\mathrm{HB}}/F_{1\mathrm{HB}}$ > 1, and as α decreases, the fraction of the motor in the 2HB state increases significantly, while if α > 1.62, the motor is in the predominant 1HB state, with $F_{2\mathrm{HB}}/F_{1\mathrm{HB}}$ < 1, and as α increases, the fraction of the motor in the 1HB state increases significantly. At small, β = 1, if α < 0.7, the motor is in the predominant 2HB state, and as α decreases, the fraction of the motor in the 2HB state increases significantly, while if α > 0.7, the motor is in the predominant 1HB state, and as α increases, the fraction of the motor in the 1HB state increases significantly. In the range of 1 < β < 100, the value of α, at which $F_{2\mathrm{HB}}/F_{1\mathrm{HB}}$ = 1, increases with the increase of β. From Figure 2b, it is seen that if α < 0.7, for any β > 1 the motor is in the predominant 2HB state, whereas if α > 1.62, for any β the motor is in the predominant 1HB state. By comparing Figure 2a with Figure 2b, it is seen interestingly that $F_{2\mathrm{HB}}/F_{1\mathrm{HB}}$ depends much more sensitively on α than on β.

The sensitive dependence of $F_{2\mathrm{HB}}/F_{1\mathrm{HB}}$ on α can be noted intuitively from the pathway of Figure 1, with the transition from Figure 1d to Figure 1f not occurring due to *P*_E_ = 1 and [ADP] = 0. It is seen that if *k*^(+)^ is evidently smaller than *k*_D_, in Figure 1e it is most probable that ADP release from the LH can occur before ATP transition to ADP in the TH. Thus, in a stepping cycle, the transition from Figure 1c to Figure 1k via Figure 1d,e,h,j can occur with an evidently larger probability than that via Figure 1d,e,g,i. Hence, the motor has a long time in the 2HB state of Figure 1e and that of Figure 1j. By contrast, if *k*^(+)^ is evidently larger than *k*_D_, in Figure 1e it is most probable that ATP transition to ADP in the TH can occur before ADP release in the LH. Thus, in a stepping cycle, the transition from Figure 1c to Figure 1k via Figure 1d,e,g,i can occur with an evidently larger probability than that via Figure 1d,e,h,j. Hence, the motor has a long time in the 1HB state of Figure 1g.

Taken together, in this section focusing on the case of saturating ATP concentrations, no free ADP, and no load, we show that in a stepping cycle the fraction of the 2HB state, *F*_2HB_, and that of the 1HB state, *F*_1HB_, are dependent only on two parameters $\alpha =k^{\left(+\right)}/k_{D}$ and $\beta =k_{\mathrm{NL}}/k_{D}$. Moreover, $F_{2\mathrm{HB}}/F_{1\mathrm{HB}}$ is determined mainly by α, while β has a minor effect on $F_{2\mathrm{HB}}/F_{1\mathrm{HB}}$. For the motor having a large *k*_NL_, if α < 1.62, the motor is in the predominant 2HB state, and as α decreases, the fraction of the 2HB state increases sensitively, while if α > 1.62, the motor is in the predominant 1HB state, and as α increases, the fraction of the 1HB state increases sensitively.

### 3.3. Effect of ATP Concentration on Fractions of 1HB and 2HB States Under No Load and No Free ADP

In this section, we study dependencies of fractions of 1HB and 2HB states on [ATP]. We consider the unloaded case, with *F* = 0 and no free ADP, with [ADP] = 0. As mentioned in the above section, we take *P*_E_ = 1 in this section. With *P*_E_ = 1 and [ADP] = 0, from Equations (3)–(12) we have the following:

(15) $$\frac{F_{2\mathrm{HB}}}{F_{1\mathrm{HB}}}=\frac{\beta \left(1+\alpha \right)}{\alpha \left[1+\alpha +\alpha \beta +B\right]},$$

(16) $$B=\frac{\alpha \beta \left(\gamma_{T}\left[\mathrm{ATP}\right]+\alpha +1\right)}{\gamma_{T}\left[\mathrm{ATP}\right]\left(\gamma_{T}\left[\mathrm{ATP}\right]+\alpha \right)}$$

where it is seen that the dependence of $F_{2\mathrm{HB}}/F_{1\mathrm{HB}}$ on [ATP] is only via the dependence of term *B* on [ATP].

From Equations (15) and (16), it is seen that for a given [ATP], besides $\alpha =k^{\left(+\right)}/k_{D}$ and $\beta =k_{\mathrm{NL}}/k_{D}$, $F_{2\mathrm{HB}}/F_{1\mathrm{HB}}$ is also dependent on $\gamma_{T}=k_{\mathrm{bT}}/k_{D}$. Prior studies showed that the rate of ADP release from the MT-bound ADP-head is about 150 s^−1^~300 s^−1^ and the second-order binding rate of ATP is about 2 μM^−1^s^−1^~5 μM^−1^s^−1^ for *Drosophila* kinesin-1 [33,38,39]. Thus, it is estimated that $\gamma_{T}=k_{\mathrm{bT}}/k_{D}$ is around 0.02 μM^−1^. Consequently, we take $\gamma_{T}$ = 0.02 μM^−1^ in this section.

**Figure 3:**
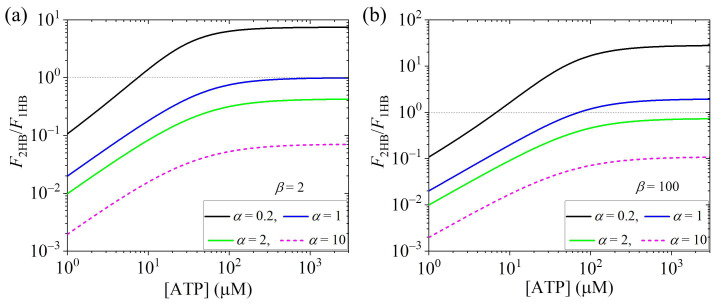
Effect of ATP concentration, [ATP], on the ratio of the fraction of the 2HB state to that of the 1HB state, $F_{2\mathrm{HB}}/F_{1\mathrm{HB}}$, under no free ADP and no load. Thin dotted lines correspond to $F_{2\mathrm{HB}}/F_{1\mathrm{HB}}$ = 1, at which the 1HB and 2HB states have the same fraction. When $F_{2\mathrm{HB}}/F_{1\mathrm{HB}}$ > 1, the motor is in the predominant 2HB state, and when $F_{2\mathrm{HB}}/F_{1\mathrm{HB}}$ < 1, the motor is in the predominant 1HB state. (**a**) $F_{2\mathrm{HB}}/F_{1\mathrm{HB}}$ vs. [ATP] for various values of α at small β = 2. (**b**) $F_{2\mathrm{HB}}/F_{1\mathrm{HB}}$ vs. [ATP] for various values of α at large β = 100.

Using Equations (15) and (16), the calculated results of $F_{2\mathrm{HB}}/F_{1\mathrm{HB}}$ vs. [ATP] for various values of α at a small β and at a large β are shown in Figure 3a and Figure 3b, respectively. It is seen that $F_{2\mathrm{HB}}/F_{1\mathrm{HB}}$ increases with the increase in [ATP] at low [ATP] and becomes leveled off at high [ATP]. Interestingly, it is seen that for a small α, the motor in the predominant 2HB at high [ATP] can become the predominant 1HB state at low [ATP]. For a large α, the motor is always in the predominant 1HB state, and the fraction of the 1HB state increases with the decrease in [ATP].

### 3.4. Effect of Load on Fractions of 1HB and 2HB States at Saturating ATP Concentrations and No Free ADP

In this section, we study dependencies of the fractions of 1HB and 2HB states on the backward load *F*. We focus on saturating ATP concentrations, with $1/\left(\gamma_{T}\left[\mathrm{ATP}\right]\right)$ approaching 0, and no free ADP, with [ADP] = 0. With $1/\left(\gamma_{T}\left[\mathrm{ATP}\right]\right)$ = 0 and [ADP] = 0, from Equations (3)–(12) we have the following:

(17) $$\frac{F_{2\mathrm{HB}}}{F_{1\mathrm{HB}}}=\frac{\beta \left[1+\alpha \left(2-P_{E}\right)+\left(1-P_{E}\right)\alpha^{2}\right]}{\alpha \left(1+\alpha +\alpha \beta P_{E}\right)}.$$

From Equations (1) and (17), it is seen that for a given *F*, besides $\alpha =k^{\left(+\right)}/k_{D}$ and $\beta =k_{\mathrm{NL}}/k_{D}$, $F_{2\mathrm{HB}}/F_{1\mathrm{HB}}$ is also dependent on *E*_0_ and δ. Here, we take *E*_0_ = 3.5 *k*_B_*T* and δ = 3.5 nm, as determined before for kinesin-1 [31].

**Figure 4:**
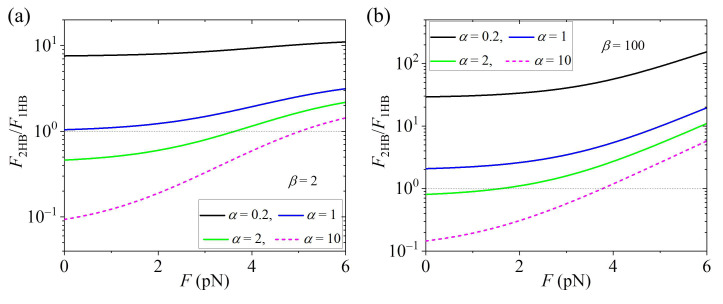
Effect of backward load, *F*, on the ratio of the fraction of 2HB state to that of 1HB state, $F_{2\mathrm{HB}}/F_{1\mathrm{HB}}$ at saturating ATP concentrations and no free ADP. Thin dotted lines correspond to $F_{2\mathrm{HB}}/F_{1\mathrm{HB}}$ = 1, at which the 1HB and 2HB states have the same fraction. When $F_{2\mathrm{HB}}/F_{1\mathrm{HB}}$ > 1, the motor is in the predominant 2HB state, and when $F_{2\mathrm{HB}}/F_{1\mathrm{HB}}$ < 1, the motor is in the predominant 1HB state. (**a**) $F_{2\mathrm{HB}}/F_{1\mathrm{HB}}$ vs. *F* for various values of α at small β = 2. (**b**) $F_{2\mathrm{HB}}/F_{1\mathrm{HB}}$ vs. *F* for various values of α at large β = 100.

Using Equations (1) and (17), the calculated results of $F_{2\mathrm{HB}}/F_{1\mathrm{HB}}$ vs. *F* for various values of α at a small β and at a large β are shown in Figure 4a and Figure 4b, respectively. It is seen that $F_{2\mathrm{HB}}/F_{1\mathrm{HB}}$ increases with the increase in *F*, especially at a large α. Interestingly, it is seen that for a large α, the motor in the predominant 1HB under no load can become the predominant 2HB state under a high load. For a small α, the motor is always in the predominant 2HB state, and the fraction of the 2HB state increases with the increase in *F*.

### 3.5. Effect of ADP Concentration on Velocity and Fractions of 1HB and 2HB States Under No Load

In this section, we study dependencies of fractions of 1HB and 2HB states on ADP concentration. We consider the unloaded case, with *F* = 0, and thus we take *P*_E_ = 1 in this section.

With *P*_E_ = 1, from Equations (5)–(13) we have the following:

(18) $$\tau =\frac{1+\alpha +C_{1}+C_{2}+C_{3}}{C_{4}}\frac{1}{k_{D}},$$

(19) $$C_{1}=\frac{\alpha \left(\gamma_{T}[\mathrm{ATP}]+\gamma_{D}[\mathrm{ADP}]+\alpha \right)+\gamma_{T}[\mathrm{ATP}]+\alpha }{\beta \left(\gamma_{T}[\mathrm{ATP}]+\gamma_{D}[\mathrm{ADP}]+\alpha \right)},$$

(20) $$C_{2}=\frac{\alpha \left(\gamma_{D}[\mathrm{ADP}]+1\right)\left(\gamma_{T}[\mathrm{ATP}]+\gamma_{D}[\mathrm{ADP}]+\alpha +1\right)}{\gamma_{T}[\mathrm{ATP}]\left(\gamma_{T}[\mathrm{ATP}]+\gamma_{D}[\mathrm{ADP}]+\alpha \right)},$$

(21) $$C_{3}=\frac{\gamma_{T}[\mathrm{ATP}]+\alpha }{\alpha \left(\gamma_{T}[\mathrm{ATP}]+\gamma_{D}[\mathrm{ADP}]+\alpha \right)},$$

(22) $$C_{4}=\frac{\alpha \left(\gamma_{T}[\mathrm{ATP}]+\gamma_{D}[\mathrm{ADP}]+\alpha +1\right)+\gamma_{T}[\mathrm{ATP}]}{\left(\gamma_{T}[\mathrm{ATP}]+\gamma_{D}[\mathrm{ADP}]+\alpha \right)}.$$

The velocity of the motor is as follows:

(23) $$v=\frac{d}{\tau },$$

where *d* = 8.3 nm is the step size, equal to the period of tubulins on a MT filament.

With *P*_E_ = 1, from Equations (3)–(12) we have the following:

(24) $$\frac{F_{2\mathrm{HB}}}{F_{1\mathrm{HB}}}=\frac{1+C_{3}}{\alpha +C_{1}+C_{2}}.$$

**Figure 5:**
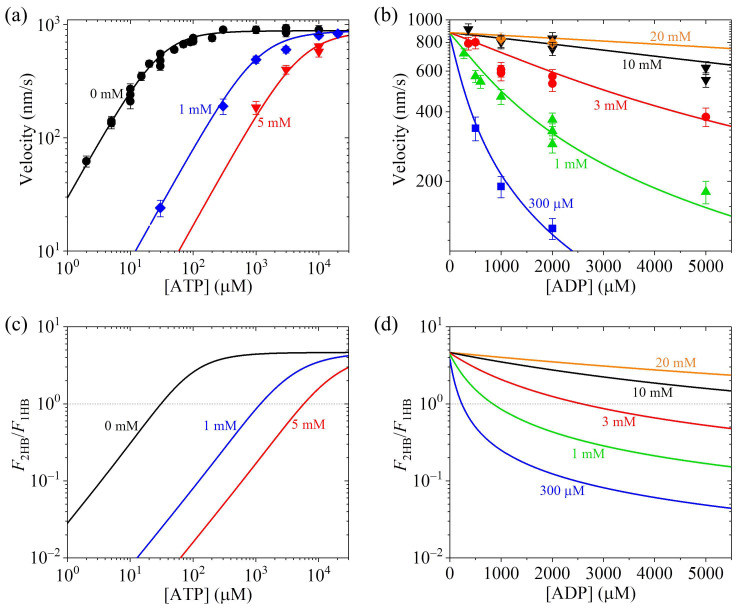
Effect of ADP concentration on velocity and ratio of the fraction of 2HB state to that of 1HB state, $F_{2\mathrm{HB}}/F_{1\mathrm{HB}}$, under no load for kinesin-1 from bovine brain. Lines are theoretical results, and symbols are experimental data from Schief et al. [40]. (**a**) Velocity vs. ATP concentration for [ADP] = 0 mM, 1 mM and 5 mM. (**b**) Velocity vs. ADP concentration for [ATP] = 300 μM, 1 mM, 3 mM, 10 mM and 20 mM. (**c**) $F_{2\mathrm{HB}}/F_{1\mathrm{HB}}$ vs. ATP concentration for [ADP] = 0 mM, 1 mM and 5 mM. (**d**) $F_{2\mathrm{HB}}/F_{1\mathrm{HB}}$ vs. ADP concentration for [ATP] = 300 μM, 1 mM, 3 mM, 10 mM and 20 mM. Thin dotted lines in (**c**,**d**) correspond to $F_{2\mathrm{HB}}/F_{1\mathrm{HB}}$ = 1, at which the 1HB and 2HB states have the same fraction. When $F_{2\mathrm{HB}}/F_{1\mathrm{HB}}$ > 1, the motor is in the predominant 2HB state, and when $F_{2\mathrm{HB}}/F_{1\mathrm{HB}}$ < 1, the motor is in the predominant 1HB state.

First, we use Equations (18)–(23) to calculate the dependencies of velocity on ATP and ADP concentrations. Since the experimental data about dependencies of velocity for kinesin-1 from bovine brain on ATP and ADP concentrations are available [40], here we compare the theoretical results with the available experimental data [40]. From Equations (18)–(23), it is noted that five parameters α, β, $\gamma_{T}$, $\gamma_{D}$ and *k*_D_ are required. To be consistent with prior biochemical data [33,37,38], we take *k*_D_ = 300 s^−1^ and *k*_NL_ = 1500 s^−1^ in this section, as achieved before for kinesin-1 [31], giving $\beta =k_{\mathrm{NL}}/k_{D}$ = 5. We take the remaining three parameters α, $\gamma_{T}$ and $\gamma_{D}$ to be adjustable. With α = 0.43, $\gamma_{T}$ = 0.012 μM^−1^ and $\gamma_{D}$ = 0.035 μM^−1^, the theoretical results reproduce quantitatively the experimental data [40] (Figure 5a,b).

Second, we use Equations (19)–(22) and (24) and parameter values presented just above to calculate the dependencies of $F_{2\mathrm{HB}}/F_{1\mathrm{HB}}$ on ATP and ADP concentrations, with the results shown in Figure 5c,d. Interestingly, it is seen that for a given ATP concentration, and especially for the ATP concentration not very large, $F_{2\mathrm{HB}}/F_{1\mathrm{HB}}$ decreases with the increase in [ADP]. For example, at [ATP] = 1 mM, $F_{2\mathrm{HB}}/F_{1\mathrm{HB}}$ > 1 when [ADP] < 0.83 mM and $F_{2\mathrm{HB}}/F_{1\mathrm{HB}}$ < 1 when [ADP] > 0.85 mM (Figure 5d). This implies that at [ATP] = 1 mM, when [ADP] < 0.83 mM the motor is in the predominant 2HB state, and when [ADP] > 0.85 mM, the motor becomes in the predominant 1HB state.

### 3.6. Applications of the Theory for Kinesin-1, Kinesin-2, Kinesin-3, Kinesin-5 and Kinesin-13 Motors

In this section, we compare the theoretical results on fractions of 1HB and 2HB states with the available experimental data for kinesin-1, kinesin-2, kinesin-3, kinesin-5 and kinesin-13 motors [13,14,15,16,17]. Since the experimental data were obtained under no load and no free ADP, we consider *F* = 0 and [ADP] = 0 here. As mentioned above, with *F* = 0, we have *P*_E_ = 1.

The ratio $F_{2\mathrm{HB}}/F_{1\mathrm{HB}}$ can be calculated by Equations (15) and (16). In a stepping cycle, the durations of the 1HB and 2HB states, which are denoted by $\tau_{1\mathrm{HB}}$ and $\tau_{2\mathrm{HB}}$, respectively, can be calculated by the following:

(25) $$\tau_{1\mathrm{HB}}=\tau F_{1\mathrm{HB}},$$

(26) $$\tau_{2\mathrm{HB}}=\tau F_{2\mathrm{HB}}.$$

Substituting Equations (2), (3) and (13) into Equations (25) and (26) and with *P*_E_ = 1 and [ADP] = 0 we obtain the following:

(27) $$\tau_{1\mathrm{HB}}=\frac{\left(1+\alpha +\alpha \beta \right)\gamma_{T}\left[\mathrm{ATP}\right]\left(\gamma_{T}\left[\mathrm{ATP}\right]+\alpha \right)+\alpha \beta \left(\gamma_{T}\left[\mathrm{ATP}\right]+\alpha +1\right)}{\gamma_{T}\left[\mathrm{ATP}\right]\left[\alpha \beta \left(\gamma_{T}\left[\mathrm{ATP}\right]+\alpha +1\right)+\gamma_{T}\left[\mathrm{ATP}\right]\right]}\frac{1}{k_{D}},$$

(28) $$\tau_{2\mathrm{HB}}=\frac{1}{\alpha k_{D}}=\frac{1}{k^{\left(+\right)}}.$$

First, we consider the kinesin-3 KIF1A motor. Prior experimental data showed that for the KIF1A dimer, ATP hydrolysis and Pi release in the TH occur very fast, with the rate being above the experimental detection limit, and the rate of ADP release from the MT-bound head has a relatively small value of about 354 s^−1^ [17]. These data indicate that *k*^(+)^ >> *k*_D_ [17], giving $\alpha =k^{\left(+\right)}/k_{D}$ >> 1. Thus, from Figure 2 it is seen that $F_{2\mathrm{HB}}/F_{1\mathrm{HB}}$ is smaller than 1, implying that at saturating ATP concentrations the KIF1A is in the predominant 1HB state. This is in accordance with the published experimental data [17].

Second, we consider kinesin-1 and kinesin-2 motors. Prior studies showed that for kinesin-1 and kinesin-2, *k*_NL_ has a value evidently larger than *k*_D_, indicating $\beta =k_{\mathrm{NL}}/k_{D}$ evidently larger than 1, and ADP release from the MT-bound ADP-head is a non-rate-limiting step of the ATPase activity, and ATP transition to ADP is the rate-limiting step [33,37,38,39], indicating $\alpha =k^{\left(+\right)}/k_{D}$ < 1. Thus, from Figure 2a it is seen that $F_{2\mathrm{HB}}/F_{1\mathrm{HB}}$ is larger than 1, implying that at saturating ATP concentrations the kinesin-1 and kinesin-2 motors are in the predominant 2HB state. This is in accordance with the published experimental data [13,14,15].

Third, we consider the kinesin-5 Eg5 motor. Prior biochemical data showed that for the Eg5 dimer, the ADP-release rate is about 76 s^−1^, the NL-docking rate is similar to the ADP-release rate, and the ATPase rate is about 8.9 s^−1^ [41], namely *k*_D_ ≈ 76 s^−1^, *k*_NL_ ≈ *k*_D_ and *k*^(+)^ ≈ 8.9 s^−1^, giving $\beta =k_{\mathrm{NL}}/k_{D}$ ≈ 1 and $\alpha =k^{\left(+\right)}/k_{D}$ ≈ 0.12. Thus, from Figure 2a it is seen that $F_{2\mathrm{HB}}/F_{1\mathrm{HB}}$ ≈ 7.5, implying that at saturating ATP concentrations the Eg5 dimer is in the predominant 2HB state. This is in accordance with the published experimental data [16].

Fourth, we consider the kinesin-13 motor. Prior studies showed that for the kinesin-13 MCAK motor on the MT lattice, ADP release is very slow [42], indicating $\alpha =k^{\left(+\right)}/k_{D}$ >> 1. Thus, from Figure 2 it is seen that $F_{2\mathrm{HB}}/F_{1\mathrm{HB}}$ << 1, implying that at saturating ATP concentrations the MCAK motor is in the predominant 1HB state on the MT lattice. This is in accordance with the previous studies showing that on the MT lattice, the MCAK motor, with only one head in the ADP state interacting with MT, makes an unbiased one-dimensional diffusion [26,27]. The experimental data of Cooper et al. [43], showing that both the MCAK monomer and dimer diffuse on the MT lattice with a similar diffusion constant, indicate also that on the MT lattice the MCAK dimer is almost in the 1HB state. By contrast, the prior studies showed that near the MT end with curved tubulins, ADP release is accelerated significantly [42], giving $\alpha =k^{\left(+\right)}/k_{D}$ << 1. Thus, from Figure 2 it is seen that $F_{2\mathrm{HB}}/F_{1\mathrm{HB}}$ >> 1, implying that near the MT end, the MCAK motor becomes in the predominant 2HB state, facilitating MT depolymerization [27].

**Figure 6:**
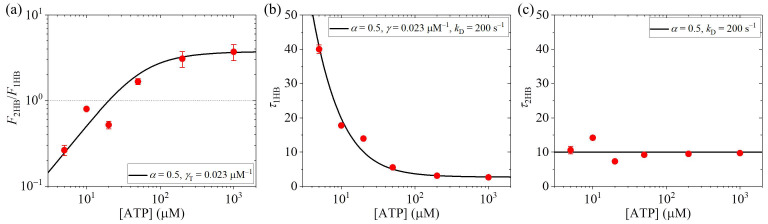
Effect of the ATP concentration, [ATP], on 1HB and 2HB states of human kinesin-1 under no load. (**a**) Ratio of the fraction of 2HB state to that of 1HB state, $F_{2\mathrm{HB}}/F_{1\mathrm{HB}}$, versus [ATP]. The thin dotted line corresponds to $F_{2\mathrm{HB}}/F_{1\mathrm{HB}}$ = 1, at which the 1HB and 2HB states have the same fraction. When $F_{2\mathrm{HB}}/F_{1\mathrm{HB}}$ > 1, the motor is in the predominant 2HB state, and when $F_{2\mathrm{HB}}/F_{1\mathrm{HB}}$ < 1, the motor is in the predominant 1HB state. (**b**) Duration of 1HB state, $\tau_{1\mathrm{HB}}$, versus [ATP]. (**c**) Duration of 2HB state, $\tau_{2\mathrm{HB}}$, versus [ATP]. In (**a**–**c**), lines are theoretical results calculated with the parameter values being indicated, and red dots are experimental data from Isojima et al. [13], with the experimental data for $F_{2\mathrm{HB}}/F_{1\mathrm{HB}}$ being calculated from those for $\tau_{1\mathrm{HB}}$ and $\tau_{2\mathrm{HB}}$.

Next, we consider non-saturating ATP concentrations for kinesin-1. Since for kinesin-1, *k*_NL_ is evidently larger than *k*_D_ [33,37,38,39] and thus $\beta =k_{\mathrm{NL}}/k_{D}$ is evidently larger than one. Hence, we fix $\beta =k_{\mathrm{NL}}/k_{D}$ = 5 here. We will adjust parameters α, $\gamma_{T}$ and *k*_D_ to make the theoretical results be consistent with the available experimental data for human kinesin-1 [13]. Using Equations (15) and (16) and by taking α = 0.5 and $\gamma_{T}$ = 0.023 μM^−1^, the theoretical results of $F_{2\mathrm{HB}}/F_{1\mathrm{HB}}$ vs. [ATP] are in good agreement with the experimental data of Isojima et al. [13], as shown in Figure 6a. Note that this value of $\gamma_{T}$ = 0.023 μM^−1^ is close to the value of about 0.02 μM^−1^ estimated above from the available biochemical data (see Section 3.2). From Figure 6a, it is evident that at high [ATP] the motor is in the predominant 2HB state and at low [ATP] the motor is in the predominant 1HB state, as noted in Section 3.2. Furthermore, using Equations (27) and (28) and additionally taking *k*_D_ = 200 s^−1^, the theoretical results of $\tau_{1\mathrm{HB}}$ and $\tau_{2\mathrm{HB}}$ vs. [ATP] are also in good agreement with the experimental data of Isojima et al. [13], as shown in Figure 6b,c. It is noted that the value of *k*_D_ = 200 s^−1^ is consistent with the available biochemical data of 150 s^−1^~300 s^−1^ [33,38,39]. The value of $\alpha =k^{\left(+\right)}/k_{D}$ = 0.5, which is determined in Figure 6a, gives *k*^(+)^ = 100 s^−1^, which is also consistent with the available biochemical data [33,38,39].

## 4. Conclusions

A theory is presented for the fractions of the 1HB and 2HB states during its processive stepping. From the theoretical results, the following conclusions are obtained:

(i) Under saturating ATP concentrations, no free ADP and no load—the fraction of the 2HB state, *F*_2HB_, and that of the 1HB state, *F*_1HB_, depend only on two parameters, $\alpha =k^{\left(+\right)}/k_{D}$, the ratio of the rate of ATP transition to ADP in the TH to the rate of ADP release from the MT-bound head, and $\beta =k_{\mathrm{NL}}/k_{D}$, the ratio of the rate of NL docking of the ATP-head to the rate of ADP release from the MT-bound head. $F_{2\mathrm{HB}}/F_{1\mathrm{HB}}$ is determined mainly by α and mildly by β. For the motor having a large *k*_NL_, when α < 1.62, the motor is in the predominant 2HB state, and as α decreases, the fraction of the 2HB state increases sensitively, while when α > 1.62, the motor is in the predominant 1HB state, and as α increases, the fraction of the 1HB state increases sensitively.
(ii) Under no free ADP and no load—$F_{2\mathrm{HB}}/F_{1\mathrm{HB}}$ increases with the increase in the ATP concentration at low ATP concentrations and becomes leveled off at high ATP concentrations. The motor having a small α, such as kinesin-1 or kinesin-2, which is in the predominant 2HB at high ATP concentrations, can become in the predominant 1HB state at low ATP concentrations. The motor having a large α, such as kinesin-3 KIF1A, is always in the predominant 1HB state, and the fraction of the 1HB state increases with the decrease in the ATP concentration.
(iii) At saturating ATP concentrations and no free ADP—$F_{2\mathrm{HB}}/F_{1\mathrm{HB}}$ increases with the increase in the backward load, especially at a large α. The motor having a large α, such as kinesin-3 KIF1A, which is in the predominant 1HB under no load, can become in the predominant 2HB state under a high load. The motor having a small α, such as kinesin-1 or kinesin-2, is always in the predominant 2HB state, and the fraction of the 2HB state increases with the increase in the backward load.
(iv) Under no load—for a given ATP concentration and, in particular, for the ATP concentration not very large, $F_{2\mathrm{HB}}/F_{1\mathrm{HB}}$ decreases with the increase in the ADP concentration. For example, for kinesin-1 at 1 mM ATP molecules, the motor is in the predominant 2HB state under no free ADP and can become in the predominant 1HB state under high ADP concentrations.

Furthermore, the theory is applied for concrete kinesin dimers such as kinesin-1, kinesin-2, kinesin-3, kinesin-5 and kinesin-13, with the theoretical results agreeing well with the published experimental data under no load. The predicted results, e.g., Figure 3 for kinesin-3 KIF1A having a large α, Figure 4 for kinesin-3 KIF1A having a large α and Figure 5c,d for kinesin-1, are hoped to be tested by future experiments.

## Figures and Tables

**Table 1:** Meanings of α, β, $\gamma_{T}$ and $\gamma_{D}$.

$\alpha =k^{\left(+\right)}/k_{D}$ Ratio of rate of ATP transition to ADP in TH to that of ADP release from MT-bound head
$\beta =k_{\mathrm{NL}}/k_{D}$ Ratio of rate of NL docking in ATP-head to that of ADP release from MT-bound head
$\gamma_{T}=k_{\mathrm{bT}}/k_{D}$ Ratio of the rate of the second-order ATP-binding rate to that of ADP release from the MT-bound head
$\gamma_{D}=k_{\mathrm{bD}}/k_{D}$ Ratio of the rate of the second-order ADP-binding rate to that of ADP release from the MT-bound head

## Data Availability

The original contributions presented in this study are included in the article.

## Supplementary Materials

The following supporting information can be downloaded at https://www.mdpi.com/article/10.3390/biom15050717/s1, Section S1: Main elements in the model for the general chemomechanical pathway of kinesin motor; Section S2: Derivation of Equations (2)–(12); Section S3: Derivation of Equation (13).
