# Supplementary material for: Factors Determining Kinesin Motors in a Predominant One-Head-Bound or Two-Heads-Bound State During Its Stepping Cycle
Source: Biomolecules. 2025 May 13;15(5):717. doi: 10.3390/biom15050717 (PMC12108896; doi:10.3390/biom15050717)
Supplement: Supplementary file 1 [file biomolecules-15-00717-s001.zip › biomolecules-3596026-supplementary.pdf]

Supplementary information

Factors determining kinesin motor in a predominant one-head-bound or two-heads-bound state during its stepping cycle

Xiao-Xuan Shi, Yu-Ru Liu, Ping Xie *

* Corresponding author: pxie@aphy.iphy.ac.cn

S1. Main elements in the model for the general chemomechanical pathway of kinesin motor

The main elements, which are consistent with the published structural, biochemical and all-atom molecular dynamics (MD) simulation evidence, are described as follows.

Interaction between kinesin head and MT — The kinesin head in nucleotide-free, ATP and ADP.Pi states has a strong interaction with or a high affinity to MT whereas in ADP state has a weak interaction with or a low affinity to MT [S1,S2]. The strong interaction of the head with MT induces large conformational changes of the local tubulin while the weak interaction induces little conformational changes, as shown by cryo-electron microscopic structural studies [S3] and all-atom molecular MD simulations [S4,S5]. Moreover, the all-atom MD simulations showed that the head in ADP state has a much weaker affinity (denoted by E_{w1}) to the tubulin having the large conformational changes than the affinity (denoted by E_{w2}) to the tubulin having no or little conformational changes [S4,S5]. On the other hand, it is expected that after Pi release from the MT-bound ADP.Pi-head there exists a short time period t_r (on the order of 10 μs) when the head has changed to the conformation in ADP state but the local tubulin is still retaining the large conformational changes and in time t_r the local tubulin returns elastically to the normally unchanged form. It is thus implied that after Pi release from the MT-bound ADP.Pi-head there exists a short time period t_r when the head has a much weaker affinity E_{w1} to the local tubulin and in time t_r the affinity of the head to the local tubulin changing from E_{w1} to E_{w2} [S6,S7]. The evidence that kinesin can induce the conformational changes of MT, which in turn can affect the MT-affinity of kinesin, is also supported by recent experimental and theoretical studies showing that the motility of a kinesin-4 motor can be affected by other kinesin motors distant away on the MT [S8–S10]. After ADP binding to the nucleotide-free head, the strong affinity of the head to MT changes to the weak affinity E_{w2}, as discussed before [S11].

Nucleotide-dependent conformation of kinesin head and its interaction with the partner head — In ADP and nucleotide-free states, the kinesin head has an open nucleotide-binding pocket (NBP) and possesses the conformation, with its flexible neck linker (NL) being unable to dock onto the head, as structural and experimental data showed [S12–S15], and the head having a high affinity to the partner ADP-head, as all-atom MD simulations showed [S16]. In ATP and ADP.Pi states, the NBP closing and a

large conformational change of the head can occur, so that NL can dock onto the head, as structural and experimental data showed [S12–S15], and the affinity of the head to the partner ADP-head can be reduced greatly, as all-atom MD simulations showed [S16]. The NL driven in the backward (minus-end-directed) orientation inhibits the NBP closing and the large conformational change of the ATP- or ADP.Pi-head.

ATPase activity of kinesin head — For most families of kinesin motors, the rate of ATP hydrolysis and Pi release in the head with the NL in the forward (plus-end-directed) orientation is much larger than that with the NL not in the forward orientation. This is supported by the following structural and experimental evidence. The structural data of the kinesin-1 head showed that the forward-directed NL clashes with a nucleotide-binding motif (a P-loop subdomain) in the nucleotide-free orientation, indicating that the P-loop subdomain is in the ATP-like orientation [S17], which would accelerate significantly the ATP hydrolysis and Pi release. This is consistent with the biochemical data showing that the kinesin-1 or kinesin-3 KIF1A head with the mutation or deletion of its NL has a much smaller ATPase rate than the wild-type case while the mutation or deletion has no effect on the rate of ADP release [S17,S18], because after ATP binding NL docks rapidly in the forward orientation. Moreover, the rate of ATP hydrolysis and Pi release of the head is independent of the force on its NL. This is in line with the experimental evidence that extending NL of each head has little effect on the ATPase rate of the kinesin-1 dimer [S19] and the ATPase rate of the truncated single kinesin-1 head is nearly equal to that of the dimer [S20–S22], because extending NL changes greatly the internal force on the NLs of the dimer [S6,S7] and no internal force is present for the single head. The evidence that the ATPase rate is independent of the force on the NL indicates that the force on the NL has no effect on the NBP. Thus, it is expected that the force also has no effect on the ATP- and ADP-binding rates to the NBP. This is consistent with the previous studies showing that by taking the second-order ATP-binding rate to be independent of the force the available single-molecule optical trapping data about dependencies of velocity, forward-to-backward stepping ratio, duration in a mechanical step, etc., on the load and ATP concentration for the kinesin-1 motors can be reproduced quantitatively [S23].

S2. Derivation of Eqs. (S2) – (S12)

In the pathway of Fig. 1, we denote by P_c, P_d, …, P_m the occurrence probabilities of states shown in Fig. 1c, d, …, m, respectively. These state probabilities at steady state can be written as

$$\frac{dP_c}{dt} = k_{bT}[\mathrm{ATP}]P_i + k^{(+)}P_j + k^{(+)}P_m - k_{NL}P_c = 0\,, \tag{S1}$$

$$\frac{dP_d}{dt} = k_{NL}P_c - k_0 P_d = 0\,, \tag{S2}$$

$$\frac{dP_e}{dt} = P_E k_0 P_d + k_{bD}[\mathrm{ADP}]P_h - \left(k^{(+)} + k_D\right)P_e = 0\,, \tag{S3}$$

$$\frac{dP_g}{dt} = k^{(+)}P_e + k_{bD}[\mathrm{ADP}]P_i - k_D P_g = 0\,, \tag{S4}$$

$$\frac{dP_{\rm h}}{dt}=k_{\rm D}P_{\rm e}-\left(k_{\rm bT}[{\rm ATP}]+k_{\rm bD}[{\rm ADP}]+k^{(+)}\right)P_{\rm h}=0\,, \tag{S5}$$

$$\frac{dP_{\rm i}}{dt}=k_{\rm D}P_{\rm g}+k^{(+)}P_{\rm h}-\left(k_{\rm bT}[{\rm ATP}]+k_{\rm bD}[{\rm ADP}]\right)P_{\rm i}=0\,, \tag{S6}$$

$$\frac{dP_{\rm j}}{dt}=k_{\rm bT}[{\rm ATP}]P_{\rm h}-k^{(+)}P_{\rm j}=0\,, \tag{S7}$$

$$\frac{dP_{\rm f}}{dt}=\left(1-P_{\rm E}\right)k_0P_{\rm d}+k_{\rm bD}[{\rm ADP}]P_{\rm l}-k_{\rm D}P_{\rm f}=0\,, \tag{S8}$$

$$\frac{dP_{\rm l}}{dt}=k_{\rm D}P_{\rm f}-\left(k_{\rm bT}[{\rm ATP}]+k_{\rm bD}[{\rm ADP}]\right)P_{\rm l}=0\,, \tag{S9}$$

$$\frac{dP_{\rm m}}{dt}=k_{\rm bT}[{\rm ATP}]P_{\rm l}-k^{(+)}P_{\rm m}=0\,, \tag{S10}$$

where k_0 represents the rate of the detached head moving from the INT position to the nearest unoccupied tubulin after the affinity of the detached head to MT-bound head becoming weak, namely the rate of transition from Fig. 1d to e or f, which is on the order of 1 $\mu{\rm s}^{-1}$. From Eqs. (S1) – (S10), we can obtain the following relationships

$$P_{\rm c}=\frac{k^{(+)}\left(k_{\rm bT}[{\rm ATP}]+k_{\rm bD}[{\rm ADP}]+k^{(+)}\right)+k_{\rm D}\left(k_{\rm bT}[{\rm ATP}]+k^{(+)}\right)}{P_{\rm E}k_{\rm NL}\left(k_{\rm bT}[{\rm ATP}]+k_{\rm bD}[{\rm ADP}]+k^{(+)}\right)}P_{\rm e}\,, \tag{S11}$$

$$P_{\rm d}=\frac{\left(k^{(+)}+k_{\rm D}\right)}{P_{\rm E}k_0}P_{\rm e}-\frac{k_{\rm bD}[{\rm ADP}]}{P_{\rm E}k_0}\frac{k_{\rm D}}{\left(k_{\rm bT}[{\rm ATP}]+k_{\rm bD}[{\rm ADP}]+k^{(+)}\right)}P_{\rm e}\,, \tag{S12}$$

$$P_{\rm f}=\frac{\left(1-P_{\rm E}\right)}{P_{\rm E}}\frac{\left(k_{\rm bT}[{\rm ATP}]+k_{\rm bD}[{\rm ADP}]\right)\left(k^{(+)}+k_{\rm D}\right)}{k_{\rm bT}[{\rm ATP}]k_{\rm D}}P_{\rm e}-\frac{\left(1-P_{\rm E}\right)}{P_{\rm E}}\frac{k_{\rm bD}[{\rm ADP}]\left(k_{\rm bT}[{\rm ATP}]+k_{\rm bD}[{\rm ADP}]\right)}{k_{\rm bT}[{\rm ATP}]\left(k_{\rm bT}[{\rm ATP}]+k_{\rm bD}[{\rm ADP}]+k^{(+)}\right)}P_{\rm e}\,, \tag{S13}$$

$$P_{\rm g}=\frac{k^{(+)}}{k_{\rm D}}P_{\rm e}+\frac{k_{\rm bD}[{\rm ADP}]k^{(+)}\left(k_{\rm bT}[{\rm ATP}]+k_{\rm bD}[{\rm ADP}]+k^{(+)}+k_{\rm D}\right)}{k_{\rm bT}[{\rm ATP}]k_{\rm D}\left(k_{\rm bT}[{\rm ATP}]+k_{\rm bD}[{\rm ADP}]+k^{(+)}\right)}P_{\rm e}\,, \tag{S14}$$

$$P_{\rm h}=\frac{k_{\rm D}}{\left(k_{\rm bT}[{\rm ATP}]+k_{\rm bD}[{\rm ADP}]+k^{(+)}\right)}P_{\rm e}\,, \tag{S15}$$

$$P_{\rm i}=\frac{k^{(+)}\left(k_{\rm bT}[{\rm ATP}]+k_{\rm bD}[{\rm ADP}]+k^{(+)}+k_{\rm D}\right)}{k_{\rm bT}[{\rm ATP}]\left(k_{\rm bT}[{\rm ATP}]+k_{\rm bD}[{\rm ADP}]+k^{(+)}\right)}P_{\rm e}\,, \tag{S16}$$

$$P_{\rm j}=\frac{k_{\rm bT}[{\rm ATP}]}{k^{(+)}}\frac{k_{\rm D}}{\left(k_{\rm bT}[{\rm ATP}]+k_{\rm bD}[{\rm ADP}]+k^{(+)}\right)}P_{\rm e}\,, \tag{S17}$$

$$P_{\rm l}=\frac{\left(1-P_{\rm E}\right)}{P_{\rm E}}\frac{\left(k^{(+)}+k_{\rm D}\right)}{k_{\rm bT}[{\rm ATP}]}P_{\rm e}-\frac{\left(1-P_{\rm E}\right)}{P_{\rm E}}\frac{k_{\rm bD}[{\rm ADP}]k_{\rm D}}{k_{\rm bT}[{\rm ATP}]\left(k_{\rm bT}[{\rm ATP}]+k_{\rm bD}[{\rm ADP}]+k^{(+)}\right)}P_{\rm e}\,, \tag{S18}$$

$$P_{\mathrm{m}} = \frac{(1-P_{\mathrm{E}})}{P_{\mathrm{E}}} \frac{\left(k^{(+)} + k_{\mathrm{D}}\right)}{k^{(+)}} P_{\mathrm{e}} - \frac{(1-P_{\mathrm{E}})}{P_{\mathrm{E}}} \frac{k_{\mathrm{bD}}[\mathrm{ADP}]k_{\mathrm{D}}}{k^{(+)}\left(k_{\mathrm{bT}}[\mathrm{ATP}] + k_{\mathrm{bD}}[\mathrm{ADP}] + k^{(+)}\right)} P_{\mathrm{e}} . \tag{S19}$$

From the pathway of Fig. 1, in a stepping cycle the fraction of the motor in 1HB state, $F_{1\mathrm{HB}}$, and that in 2HB state, $F_{2\mathrm{HB}}$, can be written as

$$F_{1\mathrm{HB}} = \frac{P_{\mathrm{c}} + P_{\mathrm{d}} + P_{\mathrm{g}} + P_{\mathrm{i}}}{P_{\mathrm{c}} + P_{\mathrm{d}} + P_{\mathrm{e}} + P_{\mathrm{f}} + P_{\mathrm{g}} + P_{\mathrm{h}} + P_{\mathrm{i}} + P_{\mathrm{j}} + P_{\mathrm{l}} + P_{\mathrm{m}}} , \tag{S20}$$

$$F_{2\mathrm{HB}} = \frac{P_{\mathrm{e}} + P_{\mathrm{f}} + P_{\mathrm{h}} + P_{\mathrm{j}} + P_{\mathrm{l}} + P_{\mathrm{m}}}{P_{\mathrm{c}} + P_{\mathrm{d}} + P_{\mathrm{e}} + P_{\mathrm{f}} + P_{\mathrm{g}} + P_{\mathrm{h}} + P_{\mathrm{i}} + P_{\mathrm{j}} + P_{\mathrm{l}} + P_{\mathrm{m}}} . \tag{S21}$$

Substituting Eqs. (S11) – (S19) into Eqs. (S20) and (S21) we have

$$F_{1\mathrm{HB}} = \frac{A_1 + A_3 + A_5}{1 + A_1 + A_2 + A_3 + A_4 + A_5 + A_6 + A_7 + A_8} , \tag{S22}$$

$$F_{2\mathrm{HB}} = \frac{1 + A_2 + A_4 + A_6 + A_7 + A_8}{1 + A_1 + A_2 + A_3 + A_4 + A_5 + A_6 + A_7 + A_8} . \tag{S23}$$

$$A_1 = \frac{k^{(+)}\left(k_{\mathrm{bT}}[\mathrm{ATP}] + k_{\mathrm{bD}}[\mathrm{ADP}] + k^{(+)}\right) + k_{\mathrm{D}}\left(k_{\mathrm{bT}}[\mathrm{ATP}] + k^{(+)}\right)}{P_{\mathrm{E}} k_{\mathrm{NL}}\left(k_{\mathrm{bT}}[\mathrm{ATP}] + k_{\mathrm{bD}}[\mathrm{ADP}] + k^{(+)}\right)} , \tag{S24}$$

$$A_2 = \frac{(1-P_{\mathrm{E}})}{P_{\mathrm{E}}} \frac{\left(k_{\mathrm{bT}}[\mathrm{ATP}] + k_{\mathrm{bD}}[\mathrm{ADP}]\right)\left(k^{(+)} + k_{\mathrm{D}}\right)}{k_{\mathrm{bT}}[\mathrm{ATP}]k_{\mathrm{D}}} - \frac{(1-P_{\mathrm{E}})}{P_{\mathrm{E}}} \frac{k_{\mathrm{bD}}[\mathrm{ADP}]\left(k_{\mathrm{bT}}[\mathrm{ATP}] + k_{\mathrm{bD}}[\mathrm{ADP}]\right)}{k_{\mathrm{bT}}[\mathrm{ATP}]\left(k_{\mathrm{bT}}[\mathrm{ATP}] + k_{\mathrm{bD}}[\mathrm{ADP}] + k^{(+)}\right)} , \tag{S25}$$

$$A_3 = \frac{k^{(+)}}{k_{\mathrm{D}}} + \frac{k_{\mathrm{bD}}[\mathrm{ADP}]k^{(+)}\left(k_{\mathrm{bT}}[\mathrm{ATP}] + k_{\mathrm{bD}}[\mathrm{ADP}] + k^{(+)} + k_{\mathrm{D}}\right)}{k_{\mathrm{bT}}[\mathrm{ATP}]k_{\mathrm{D}}\left(k_{\mathrm{bT}}[\mathrm{ATP}] + k_{\mathrm{bD}}[\mathrm{ADP}] + k^{(+)}\right)} , \tag{S26}$$

$$A_4 = \frac{k_{\mathrm{D}}}{\left(k_{\mathrm{bT}}[\mathrm{ATP}] + k_{\mathrm{bD}}[\mathrm{ADP}] + k^{(+)}\right)} , \tag{S27}$$

$$A_5 = \frac{k^{(+)}\left(k_{\mathrm{bT}}[\mathrm{ATP}] + k_{\mathrm{bD}}[\mathrm{ADP}] + k^{(+)} + k_{\mathrm{D}}\right)}{k_{\mathrm{bT}}[\mathrm{ATP}]\left(k_{\mathrm{bT}}[\mathrm{ATP}] + k_{\mathrm{bD}}[\mathrm{ADP}] + k^{(+)}\right)} , \tag{S28}$$

$$A_6 = \frac{k_{\mathrm{bT}}[\mathrm{ATP}]}{k^{(+)}} \frac{k_{\mathrm{D}}}{\left(k_{\mathrm{bT}}[\mathrm{ATP}] + k_{\mathrm{bD}}[\mathrm{ADP}] + k^{(+)}\right)} , \tag{S29}$$

$$A_7 = \frac{(1-P_{\mathrm{E}})}{P_{\mathrm{E}}} \frac{\left(k^{(+)} + k_{\mathrm{D}}\right)}{k_{\mathrm{bT}}[\mathrm{ATP}]} - \frac{(1-P_{\mathrm{E}})}{P_{\mathrm{E}}} \frac{k_{\mathrm{bD}}[\mathrm{ADP}]k_{\mathrm{D}}}{k_{\mathrm{bT}}[\mathrm{ATP}]\left(k_{\mathrm{bT}}[\mathrm{ATP}] + k_{\mathrm{bD}}[\mathrm{ADP}] + k^{(+)}\right)} , \tag{S30}$$

$$A_8 = \frac{(1-P_{\mathrm{E}})}{P_{\mathrm{E}}} \frac{\left(k^{(+)} + k_{\mathrm{D}}\right)}{k^{(+)}} - \frac{(1-P_{\mathrm{E}})}{P_{\mathrm{E}}} \frac{k_{\mathrm{bD}}[\mathrm{ADP}]k_{\mathrm{D}}}{k^{(+)}\left(k_{\mathrm{bT}}[\mathrm{ATP}] + k_{\mathrm{bD}}[\mathrm{ADP}] + k^{(+)}\right)} , \tag{S31}$$

where we have implicitly considered that k_0 is infinitely large.

Defining $k^{(+)} = \alpha k_{\mathrm{D}}$, $k_{\mathrm{NL}} = \beta k_{\mathrm{D}}$, $k_{\mathrm{bT}} = \gamma_{\mathrm{T}} k_{\mathrm{D}}$ and $k_{\mathrm{bD}} = \gamma_{\mathrm{D}} k_{\mathrm{D}}$, where α and β are dimensionless

constants while γ_{T} and γ_{D} are constants in units of μM^{-1}, Eqs. (S24) – (S31) can be rewritten as

$$A_1 = \frac{\alpha(\gamma_{\mathrm{T}}[\mathrm{ATP}]+\gamma_{\mathrm{D}}[\mathrm{ADP}]+\alpha)+\gamma_{\mathrm{T}}[\mathrm{ATP}]+\alpha}{P_{\mathrm{E}}\beta(\gamma_{\mathrm{T}}[\mathrm{ATP}]+\gamma_{\mathrm{D}}[\mathrm{ADP}]+\alpha)}, \tag{S32}$$

$$A_2 = \frac{(1-P_{\mathrm{E}})}{P_{\mathrm{E}}}\frac{(\gamma_{\mathrm{T}}[\mathrm{ATP}]+\gamma_{\mathrm{D}}[\mathrm{ADP}])(\alpha+1)}{\gamma_{\mathrm{T}}[\mathrm{ATP}]} - \frac{(1-P_{\mathrm{E}})}{P_{\mathrm{E}}}\frac{\gamma_{\mathrm{D}}[\mathrm{ADP}](\gamma_{\mathrm{T}}[\mathrm{ATP}]+\gamma_{\mathrm{D}}[\mathrm{ADP}])}{\gamma_{\mathrm{T}}[\mathrm{ATP}](\gamma_{\mathrm{T}}[\mathrm{ATP}]+\gamma_{\mathrm{D}}[\mathrm{ADP}]+\alpha)}, \tag{S33}$$

$$A_3 = \alpha + \frac{\alpha\gamma_{\mathrm{D}}[\mathrm{ADP}](\gamma_{\mathrm{T}}[\mathrm{ATP}]+\gamma_{\mathrm{D}}[\mathrm{ADP}]+\alpha+1)}{\gamma_{\mathrm{T}}[\mathrm{ATP}](\gamma_{\mathrm{T}}[\mathrm{ATP}]+\gamma_{\mathrm{D}}[\mathrm{ADP}]+\alpha)}, \tag{S34}$$

$$A_4 = \frac{1}{(\gamma_{\mathrm{T}}[\mathrm{ATP}]+\gamma_{\mathrm{D}}[\mathrm{ADP}]+\alpha)}, \tag{S35}$$

$$A_5 = \frac{\alpha(\gamma_{\mathrm{T}}[\mathrm{ATP}]+\gamma_{\mathrm{D}}[\mathrm{ADP}]+\alpha+1)}{\gamma_{\mathrm{T}}[\mathrm{ATP}](\gamma_{\mathrm{T}}[\mathrm{ATP}]+\gamma_{\mathrm{D}}[\mathrm{ADP}]+\alpha)}, \tag{S36}$$

$$A_6 = \frac{\gamma_{\mathrm{T}}[\mathrm{ATP}]}{\alpha(\gamma_{\mathrm{T}}[\mathrm{ATP}]+\gamma_{\mathrm{D}}[\mathrm{ADP}]+\alpha)}, \tag{S37}$$

$$A_7 = \frac{(1-P_{\mathrm{E}})}{P_{\mathrm{E}}}\frac{(\alpha+1)}{\gamma_{\mathrm{T}}[\mathrm{ATP}]} - \frac{(1-P_{\mathrm{E}})}{P_{\mathrm{E}}}\frac{\gamma_{\mathrm{D}}[\mathrm{ADP}]}{\gamma_{\mathrm{T}}[\mathrm{ATP}](\gamma_{\mathrm{T}}[\mathrm{ATP}]+\gamma_{\mathrm{D}}[\mathrm{ADP}]+\alpha)}, \tag{S38}$$

$$A_8 = \frac{(1-P_{\mathrm{E}})}{P_{\mathrm{E}}}\frac{(\alpha+1)}{\alpha} - \frac{(1-P_{\mathrm{E}})}{P_{\mathrm{E}}}\frac{\gamma_{\mathrm{D}}[\mathrm{ADP}]}{\alpha(\gamma_{\mathrm{T}}[\mathrm{ATP}]+\gamma_{\mathrm{D}}[\mathrm{ADP}]+\alpha)}. \tag{S39}$$

S3. Derivation of Eq. (S13)

From the pathway of Fig. 1, the duration of a mechanical step (i.e., the transition from Fig. 1c to k) can be written as

$$\tau = \frac{P_{\mathrm{c}}+P_{\mathrm{d}}+P_{\mathrm{e}}+P_{\mathrm{f}}+P_{\mathrm{g}}+P_{\mathrm{h}}+P_{\mathrm{i}}+P_{\mathrm{j}}+P_{\mathrm{l}}+P_{\mathrm{m}}}{k_{\mathrm{bT}}[\mathrm{ATP}]P_{\mathrm{i}}+k^{(+)}P_{\mathrm{j}}}. \tag{S40}$$

Substituting Eqs. (S11) – (S19) into Eq. (S40) and with the definition of $k^{(+)}=\alpha k_{\mathrm{D}}$, $k_{\mathrm{NL}}=\beta k_{\mathrm{D}}$, $k_{\mathrm{bT}}=\gamma_{\mathrm{T}}k_{\mathrm{D}}$ and $k_{\mathrm{bD}}=\gamma_{\mathrm{D}}k_{\mathrm{D}}$ we finally obtain

$$\tau = \frac{1+A_1+A_2+A_3+A_4+A_5+A_6+A_7+A_8}{\gamma_{\mathrm{T}}[\mathrm{ATP}]A_5+\alpha A_6}\frac{1}{k_{\mathrm{D}}}. \tag{S41}$$

where we have implicitly considered that k_0 is infinitely large.

References

[S1] Crevel I.M.T.C., Lockhart A., Cross R.A. (1996) Weak and strong states of kinesin and Ncd. J. Mol. Biol. 257, 66-76.

[S2] Sosa H., et al. (2001) ADP-induced rocking of the kinesin motor domain revealed by single-molecule fluorescence polarization microscopy. Nat. Struc. Biol. 8, 540-544.

[S3] Morikawa M., et al. (2015) X-ray and Cryo-EM structures reveal mutual conformational changes of kinesin and GTP-state microtubules upon binding. EMBO J. 34, 1270−1286.

[S4] Shi X.-X., et al. (2018) Investigating role of conformational changes of microtubule in regulating its binding affinity to kinesin by all-atom molecular dynamics simulation. Proteins 86, 1127–1139.

[S5] Shi X.-X., et al. (2021) Studies of conformational changes of tubulin induced by interaction with kinesin using atomistic molecular dynamics simulations. Int. J. Mol. Sci. 22, 6709.

[S6] Guo S.-K., et al. (2017) A model of processive movement of dimeric kinesin. J. Theor. Biol. 414, 62-75.

[S7] Guo S.-K., et al. (2018) Processivity of dimeric kinesin-1 molecular motors. FEBS Open Bio **8**: 1332–1351.

[S8] Verhey K.J., Ohi R. (2023) Causes, costs and consequences of kinesin motors communicating through the microtubule lattice. J. Cell Sci. 136, jcs260735.

[S9] Wijeratne S.S., et al. (2022). Motor guidance by long-range communication on the microtubule highway. Proc. Natl. Acad. Sci. U.S.A. 119, e2120193119.

[S10] Xie P. (2024) A model for cooperativity of kinesin-4 motors by communicating through the microtubule track. Chem. Phys. 581, 112274.

[S11] Xie P. (2021) Modeling processive motion of kinesin-13 MCAK and kinesin-14 Cik1-Kar3 molecular motors. Protein Sci. 30, 2092–2105.

[S12] Shang Z., et al (2014) High-resolution structures of kinesin on microtubules provide a basis for nucleotide-gated force-generation. eLife 3, e04686.

[S13] Sindelar C.V., Downing K.H. (2010) An atomic-level mechanism for activation of the kinesin molecular motors. Proc. Natl. Acad. Sci. U.S.A. 107, 4111–4116.

[S14] Rice S., et al. (1999) A structural change in the kinesin motor protein that drives motility. Nature 402, 778–784.

[S15] Asenjo A.B., Weinberg Y., Sosa H. (2006) Nucleotide binding and hydrolysis induces a disorder-order transition in the kinesin neck-linker region. Nat. Struc. Mol. Biol. 13, 648–654.

[S16] Shi X.-X., et al. (2020) All-atom molecular dynamics simulations reveal how kinesin transits from one-head-bound to two-heads-bound state. Proteins 88, 545–557.

[S17] Cao L., et al. (2014) The structure of apo-kinesin bound to tubulin links the nucleotide cycle to movement. Nat. Commun. 5, 5364.

[S18] Nitta R., Okada Y., Hirokawa N. (2008) Structural model for strain-dependent microtubule activation of Mg-ADP release from kinesin. Nat. Struct. Mol. Biol. 15, 1067–1075.

[S19] Yildiz A., et al. (2008) Intramolecular strain coordinates kinesin stepping behavior along microtubules. Cell 134, 1030-1041.

[S20] Moyer M.L., Gilbert S.P., Johnson K.A. (1998) Pathway of ATP hydrolysis by monomeric and dimeric kinesin. Biochemistry 37, 800-813.

[S21] Okada Y., Hirokawa N. (1999) A processive single-headed motor: kinesin superfamily protein KIF1A. Science 283, 1152–1157.

[S22] Rosenfeld S.S., et al. (2003) Stepping and stretching: how kinesin uses internal strain to walk processively. J. Biol. Chem. 278, 18550–18556.

[S23] Xie P., Guo S., Chen H. (2019) ATP-concentration- and force-dependent chemomechanical coupling of kinesin molecular motors. J. Chem. Inf. Model. 59, 360−372.
